# Ability of the post-operative ALBI grade to predict the outcomes of hepatocellular carcinoma after curative surgery

**DOI:** 10.1038/s41598-020-64354-0

**Published:** 2020-04-29

**Authors:** Wei-Ru Cho, Chao-Hung Hung, Chien-Hung Chen, Chih-Che Lin, Chih-Chi Wang, Yueh-Wei Liu, Yi-Ju Wu, Chee-Chien Yong, Kuang-Den Chen, Yu-Chieh Tsai, Tsung-Hui Hu, Ming-Chao Tsai

**Affiliations:** 1 Division of Hepato-Gastroenterology, Department of Internal Medicine, Kaohsiung Chang Gung Memorial Hospital and Chang Gung University College of Medicine, Kaohsiung, Taiwan; 2 Liver Transplantation Center and Department of Surgery, Kaohsiung Chang Gung Memorial Hospital and Chang Gung University College of Medicine, Kaohsiung, Taiwan; 3 Center for Translational Research in Biomedical Sciences, Liver Transplantation Program and Department of Surgery, Kaohsiung Chang Gung Memorial Hospital and Chang Gung University College of Medicine, Kaohsiung, Taiwan; 4 Department of Internal Medicine, Kaohsiung Chang Gung Memorial Hospital, Kaohsiung, Taiwan; 5 Graduate Institute of Clinical Medical Sciences, Chang Gung University College of Medicine, Kaohsiung, Taiwan

**Keywords:** Cancer prevention, Hepatocytes

## Abstract

The albumin-bilirubin (ALBI) grade has been validated as a significant predictor for hepatocellular carcinoma (HCC). However, there is little information about the impact of postoperative ALBI grade in patients with HCC who are undergoing liver resection. We enrolled 525 HCC patients who received primary resection from April 2001 to March 2017. The impact of the pre- and post-operative ALBI grades on overall survival (OS) and recurrence-free survival (RFS) were analyzed by multivariate analysis. During the follow-up period (mean, 65 months), 253 (48.1%) patients experienced recurrence, and 85 (16.2%) patients died. Multivariate analysis revealed that diabetes mellitus (DM) (*p* = 0.011), alpha-fetoprotein levels (AFP) (*p* < 0.001), low platelet count (*p* = 0.008), liver cirrhosis (*p* < 0.001), and the first year of ALBI grade after resection (*p* < 0.001) were independent predictors for RFS. Additionally, old age (*p* = 0.006), DM (*p* = 0.002), AFP (*p* = 0.027), and ALBI grade at the first year after resection (*p* < 0.001) were independent risk factors for poor liver-related survival. Patients with post-operative ALBI grades II/III had older age (p = 0.019), hypoalbuminemia (p = 0.038), DM (p = 0.043), and high stages of pTNM (p = 0.021). The post-operative ALBI grade is better for predicting the outcomes in HCC patients after curative hepatectomy than the pre-operative ALBI grade.

## Introduction

Hepatocellular carcinoma (HCC) is the fifth most common cancer worldwide, and its incidence is approximately 850,000 new cases per year^1,2^. HCC is often considered to be linked to multiple risk factors, such as infections with hepatitis B virus (HBV) or hepatitis C virus (HCV), alcohol abuse, and metabolic syndrome^3^. In North America, Europe, and Japan, HCV is the leading cause of HCC, while in many Asian countries, such as Taiwan, HCC is most frequently associated with chronic HBV infection^4^.

To date, hepatectomy remains the most effective treatment for patients with early-stage HCC who have well-preserved liver function. Nevertheless, the overall survival (OS) after curative resection remains unsatisfactory because of the high rate of recurrence. The 5-year survival rate after resection is up to 50–70%^5–7^. The prognosis and management of HCC depend on the tumor stage, the performance status, and the patient’s liver reserve. Therefore, assessment of the liver’s functional reserve is an integral part of HCC management.

Recently, there is a novel evaluation model called the albumin-bilirubin (ALBI) grade, which exhibited impressive performance for predicting the prognosis of HCC patients^8^. The model is composed of two routine clinical tests (albumin and bilirubin tests) and can stratify patients with HCC into three risk categories. Patients with different ALBI grades have different OS. Moreover, patients with the same Child-Pugh score experience different OS significantly when stratified according to ALBI grade. This indicates that the ALBI grade might provide better prognostic performance compared with the Child-Pugh score for predicting the OS of HCC patients.

Previous studies have demonstrated that the ALBI grade could be used to discriminate patient survival, but there is little information in regard to the impact of postoperative ALBI grade in cases of HCC after resection. Therefore, in the present study, we evaluated the effect of the pre- and post-operative ALBI grades in predicting the outcomes of patients with HBV-related HCC after curative hepatectomy.

## Results

### Patient characteristics

Table 1 presents the baseline characteristics of the study cohort. The mean follow-up time was 65 months. The sample comprised 445 men and 80 women, and the median age was 54 years at enrollment. According to the pre-operative Child-Pugh system, the majority of patients were grade A (96%, 503/525). As shown in Table 1, there were 224 (43%) patients who were stratified into pre-operative ALBI grade I and 301 (57%) who were stratified into ALBI grade II, but none were in ALBI grade III. Compared to patients with pre-operative ALBI grade I, patients with pre-operative ALBI grade II were significantly older (p < 0.001) and had a higher percentage of cirrhosis (p = 0.02), Child-Pugh grade B (p = 0.018), high pTNM stage (p = 0.021), higher serum bilirubin (p < 0.001), FIB-4 score (p < 0.001), and lower serum albumin levels (p = 0.038). Overall, patients with pre-operative ALBI grade II had higher rates of death (19.6%) than subjects with pre-operative ALBI grade I (11.6%, p = 0.014).

**Table 1:** Comparison of clinical and pathological characteristics between patients with pre-operative ALBI grades I and II.

	Total (n = 525)	ALBI grade I (n = 224)	ALBI grade II (n = 301)	P value
Age (years; mean ± SD)	53.5 ± 11.2	51.4 ± 10.9	55.1 ± 11.2	<0.001
Age (>60 years), n (%)	179 (34.1%)	52 (23.2%)	127 (42.2%)	<0.001
Male, n (%)	445 (84.8%)	189 (84.4%)	256 (85%)	0.831
Bilirubin (g/dL; mean ± SD)	0.9 ± 0.3	0.8 ± 0.3	0.9 ± 0.4	<0.001
Albumin (g/dL; mean ± SD)	3.8 ± 0.6	4.3 ± 0.3	3.5 ± 0.4	0.038
AFP (>15 ng/mL), n (%)	259 (50.3%)	114 (51.4%)	145 (49.5%)	0.675
Liver cirrhosis, n (%)	244 (46.5%)	91 (40.6%)	153 (50.8%)	0.020
HBV DNA (≥20000 IU/ml)	141 (26.4%)	57 (25.4%)	84 (27.9%)	0.539
NAs treatment, n (%)	150 (28.6%)	65 (29%)	85 (28.2%)	0.845
LAM, n (%)	28 (18.7%)	16 (24.6%)	12 (14.1%)	
LdT, n (%)	9 (6%)	5 (7.7%)	4 (4.7%)	
ETV, n (%)	96 (64%)	37 (56.9%)	59 (69.4%)	
TDF, n (%)	17 (11.3%)	7 (10.8%)	10 (11.8%)	
Tumor size (>5 cm), n (%)	96 (18%)	43 (19.2%)	51 (17%)	0.517
Tumor number (single: multiple)	499: 26	213: 11	286: 15	0.970
Types of surgery				0.853
Right lobectomy, n (%)	101 (19.2%)	42 (18.8%)	59 (19.6%)	
Left lobectomy, n (%)	61 (11.6%)	28 (12.5%)	33 (11%)	
Segmentectomy, n (%)	363 (69.1%)	154 (68.8%)	209 (69.4%)	
Child-Pugh grade (A: B)	503: 22	220: 4	283: 18	0.018
MELD score (mean ± SD)	8.0 ± 3.1	7.9 ± 3.4	8.0 ± 2.9	0.813
FIB-4 score	2.3 ± 1.7	1.9 ± 1.2	2.6 ± 2.0	<0.001
Diabetes Mellitus	78 (15.6%)	35 (15.4%)	43 (14.6%)	0.804
cTNM stage (I: II)	280: 204	122: 83	158: 121	0.526
pTNM stage (I: II: III)	280: 204: 41	88: 78: 5	31: 27: 8	0.021
Micro/Macrovascular invasion, n (%)	218 (41.5%)	88 (39.3%)	130 (43.2%)	0.369
Histological grade (well: moderate: poor)	69: 440: 16	32: 187: 5	37: 253: 11	0.536
Recurrence, n (%)	247 (47%)	100 (44.6%)	147 (48.8%)	0.341
Death, n (%)	85 (16.2%)	26 (11.6%)	59 (19.6%)	0.014

AFP = α-fetoprotein, NAs, = nucleot(s)ide analogues, LAM = lamivudine, LdT = telbivudine, ETV = entecavir, TDF = tenofovir, MELD = model for end-stage liver disease, FIB-4 = fibrosis-4, ALBI = albumin-bilirubin.

### Pre-operative ALBI grades correlate with the survival of HCC patients after curative resection

After a median follow-up of 65 months, 253 patients (48.1%) had recurrent HCCs, and 88 (16.5%) died. Of those who died, 82 suffered from liver-related death, including 70 due to HCC and 12 from complications associated with cirrhosis. Of the 6 patients with non-liver-related death, 4 died from severe infection, and 2 died from malignancies other than HCC.

**Figure 1:**
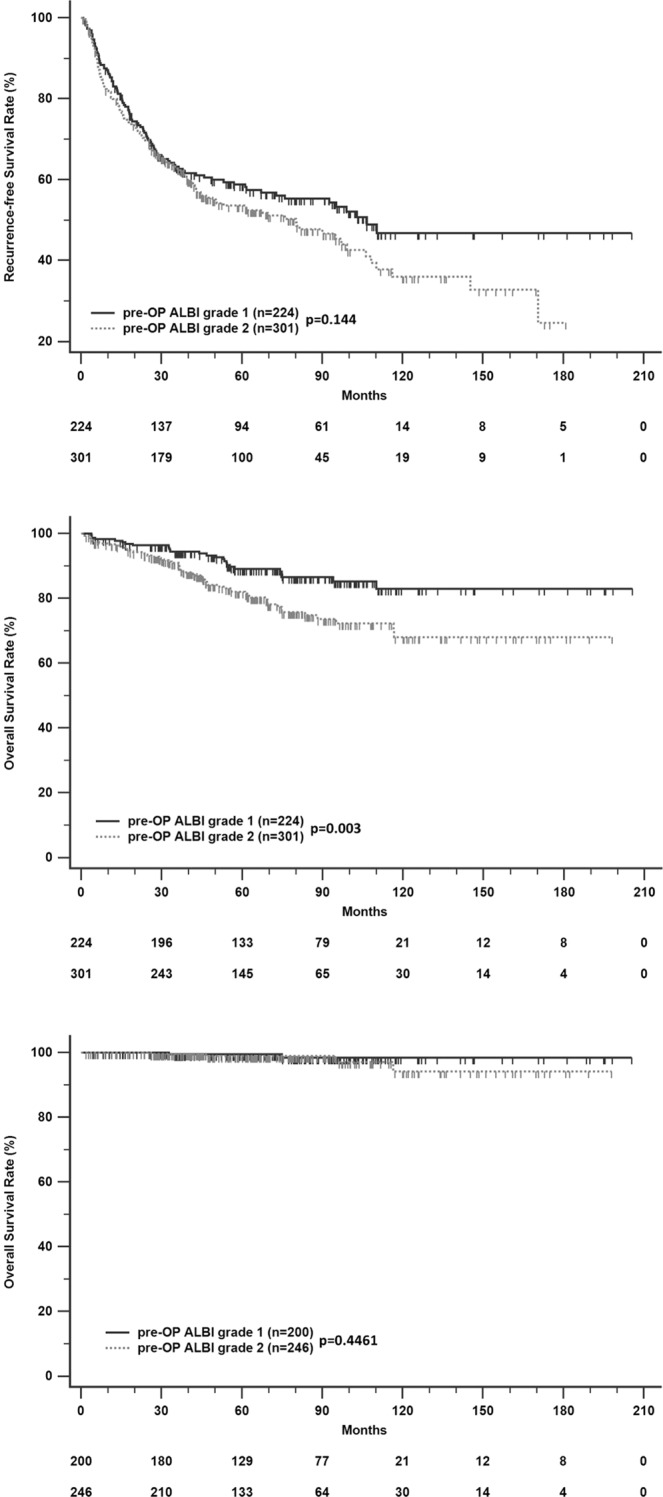
Recurrence-free survival (**A**), liver-related survival (**B**), and non-liver-related survival (**C**) in HCC patients after curative resection according to stratified pre-operative ALBI grade.

We first investigated the predictive value of the pre-operative ALBI grade for all subjects. The liver-related survival of patients with pre-operative ALBI grade II was significantly shorter than that of patients with ALBI grade I (p = 0.003) (Fig. 1B), but no significance was noted in non-liver-related survival (Fig. 1C). In the RFS analysis, there were no significant differences between patients with ALBI grades I and II, but patients with pre-operative ALBI grade I showed a greater RFS rate than those with ALBI grade II (Fig. 1A).

### Post-operative ALBI grades correlate with the recurrence and survival of HCC patients after curative resection

**Figure 2:**
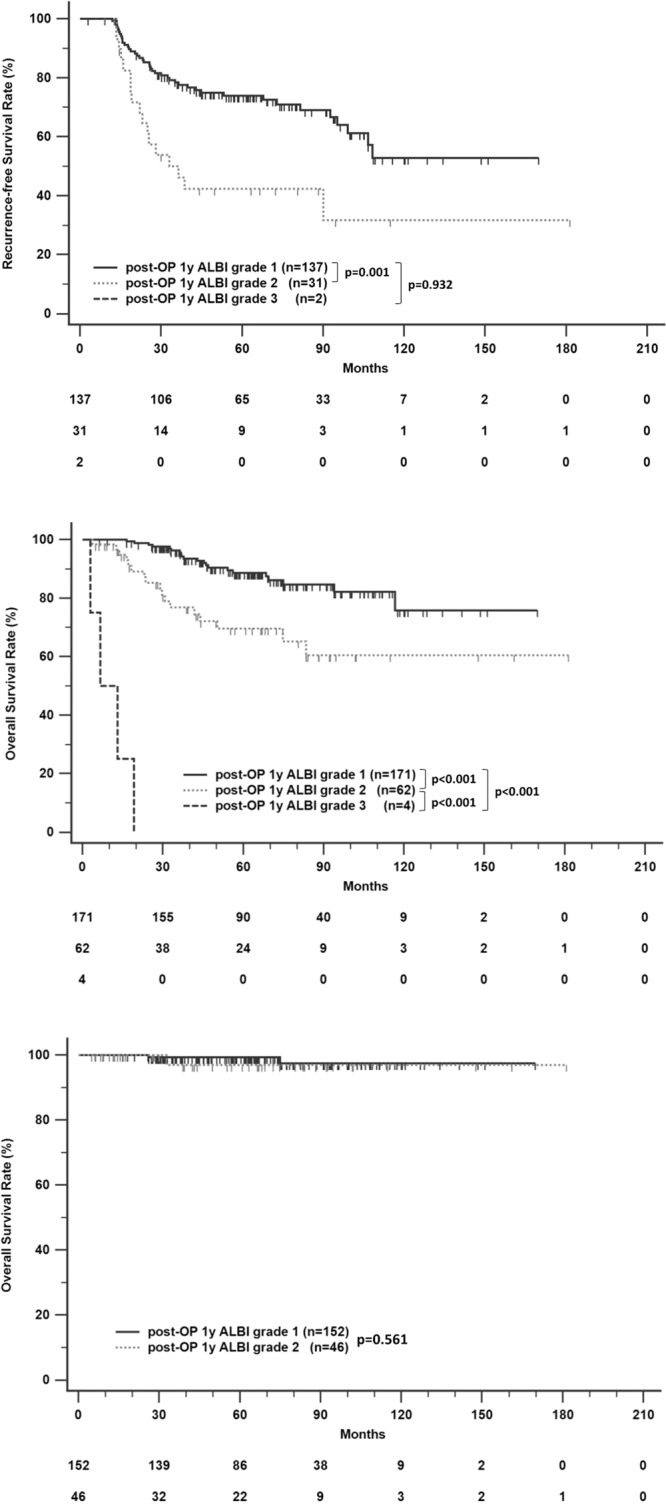
Recurrence-free survival (**A**), liver-related survival (**B**), and non-liver-related survival (**C**) in HCC patients after curative resection according to stratified ALBI grade at 1 year post-operation.

We also investigated the predictive value of the post-operative ALBI grade. We excluded 95 patients who had recurrence within the first year after resection. The group with ALBI grade I at the first year post-operation had better RFS than patients with ALBI grades II (p = 0.001) and III (p < 0.001) (Fig. 2A). The liver-related survival of patients with ALBI grades II and III was significantly shorter than those of patients with ALBI grade I (p < 0.0001, both) (Fig. 2B). There was no significant difference in non-liver-related death among those with different post-operative ALBI grades (Fig. 2C).

### Independent factors for recurrence free survival and OS of HCC patients after curative resection

As shown in Table 2, age, diabetes mellitus (DM), thrombocytopenia, hypoalbuminemia, liver cirrhosis, TNM stage, histology stage, vascular invasion, post-operative first year albumin and ALBI grade were significantly associated with RFS. In the multivariate analysis, older age (hazard ratio [HR], 1.585; 95% CI, 1.038-2.42; *p* = 0.033), AFP > 15 ng/mL (HR, 3; 95% CI, 2.049-4.392; *p* < 0.001), DM (HR, 1.802; 95% CI, 1.144-2.838; *p* = 0.011), thrombocytopenia (HR, 1.85; 95% CI, 1.176-2.911; *p* = 0.008), liver cirrhosis (HR, 2.267; 95% CI, 1.504-3.417; *p* < 0.001), and ALBI grade II/III at 1 year post-operation (HR, 3.0; 95% CI, 2.049-4.392; *p* < 0.001) remained independent predictive factors for RFS.

**Table 2:** Univariate and multivariate analysis for recurrence in CHB patients with HCC after curative hepatectomy.

Variable	Comparison	Univariate		Multivariate	
		HR (95%CI)	P value	HR (95%CI)	P value
Age (years)	>60 vs. ≦ 60	1.345 (1.042–1.736)	0.023	1.585 (1.038–2.420)	0.033
Sex	Male vs. Female	0.970 (0.689–1.364)	0.860		
DM	Yes vs. No	2.036 (1.493–2.778)	<0.001	1.802 (1.144–2.838)	0.011
AFP (ng/mL)	>15 vs. ≦ 15	1.264 (0.983–1.625)	0.067	3.000 (2.049 – 4.392)	<0.001
Platelet (10^9^/L)	≦ 150 vs. >150	1.408 (1.091–1.816)	0.009	1.850 (1.176–2.911)	0.008
Albumin (g/dL)	≦ 3.5 vs. >3.5	1.328 (1.010–1.746)	0.042		
Liver cirrhosis	Yes vs. No	2.073 (1.612–2.668)	<0.001	2.267 (1.504–3.417)	<0.001
HBV DNA (IU/ml)	>2000 vs. ≦ 2000	1.074 (0.825–1.237)	0.732		
NAs treatment	No vs. Yes	1.925 (0.894–2.269)	0.076		
MELD score	>14 vs. ≦ 14	0.967 (0.430 - 2.174)	0.935		
Child-Pugh grade	B vs. A	1.400 (0.764–2.563)	0.276		
Types of surgery	Segmentectomy	ref			
	Left lobectomy	1.139 (0.776–1.672)	0.505		
	Right lobectomy	1.075 (0.783–1.475)	0.656		
Pre-operative ALBI grade	II vs. I	1.209 (0.937–1.559)	0.145		
Tumor no.	Multiple vs. Single	1.395 (0.840–2.316)	0.198		
Tumor size (cm)	>5 vs. ≦ 5	1.372 (1.018–1.849)	0.038		
pTNM stages	III vs. I + II	1.599 (1.072–2.385)	0.021		
Histology stages	poor vs. well + moderate	1.893 (1.005–3.565)	0.048		
Vascular invasion	Yes vs. No	1.536 (1.197–1.970)	0.001		
Post-operative first year albumin (g/dL)	≦ 3.5 vs. >3.5	2.723 (1.757–4.536)	<0.001		
Post-operative first year ALBI grade	II/III vs. I	2.906 (2.022–4.178)	<0.001	3.000 (2.049–4.392)	<0.001

AFP = α-fetoprotein, NAs, = nucleot(s)ide analogues, MELD = model for end-stage liver disease, FIB-4 = fibrosis-4, ALBI = albumin-bilirubin.

In the OS analysis, the multivariate Cox proportional hazards model revealed that older age (HR, 2.586; 95% CI, 1.311-5.098; *p* = 0.006), DM (HR, 2.751; 95% CI, 1.432-5.283; *p* = 0.002), AFP > 15 ng/mL (HR, 2.197; 95% CI, 1.095-4.409; *p* = 0.027), pTNM staging (HR, 2.064; 95% CI, 1.071-3.978; *p* = 0.03), and ALBI grade II/III at the first year post-operation (HR, 3.246; 95% CI, 1.726-6.105; *p* < 0.001) were independent risk factors associated with overall mortality. In regard to liver-related death, older age (HR, 2.831; 95% CI, 1.415-5.663; *p* = 0.003), DM (HR, 2.316; 95% CI, 1.173-4.576; *p* = 0.016), AFP > 15 ng/mL (HR, 2.261; 95% CI, 1.114-4.588; *p* = 0.024), pTNM staging (HR, 2.025; 95% CI, 1.034-3.966; *p* = 0.04), and ALBI grade II/III at the first year post-operation (HR, 3.585; 95% CI, 1.874-6.855; *p* < 0.001) remained independent risk factors for survival (Table 3).

**Table 3:** Multivariate analysis for overall and liver-related survival in CHB patients with HCC after curative hepatectomy.

Variable	Comparison	Overall survival		Liver-related death	
		HR (95%CI)	P value	HR (95%CI)	P value
Age (years)	>60 vs. ≦ 60	2.586 (1.311–5.098)	0.006	2.831 (1.415–5.663)	0.003
DM	Yes vs. No	2.751 (1.432–5.283)	0.002	2.316 (1.173–4.576)	0.016
AFP (ng/mL)	>15 vs. ≦ 15	2.197 (1.095–4.409)	0.027	2.261 (1.114–4.588)	0.024
pTNM stages	II/III vs. I	2.064 (1.071–3.978)	0.030	2.025 (1.034–3.966)	0.040
Post-operative first year ALBI grade	II/III vs. I	3.246 (1.726–6.105)	<0.001	3.585 (1.874–6.855)	<0.001

Abbreviations: HR: hazard ratio; CI: confidence interval; DM: diabetes mellitus, AFP: α-fetoprotein; ALBI: albumin-bilirubin.

### Association between post-operative ALBI grade and patient characteristics

There were 66 patients (27.8%) who had ALBI grade II/III at the first year after curative resection. Among them, four patients were categorized into ALBI grade III and all of them had liver cirrhosis. We also analyzed the association between post-operative ALBI grade and pre-operative characteristics. Table 4 shows the association between ALBI grade at the first year post-operation (II/III vs. I) and pre-operative characteristics. Patients with post-operative ALBI grade II/III were older (p = 0.019) and had lower levels of albumin (p = 0.038), a higher rate of DM (p = 0.043), and a higher stage of pTNM (p = 0.021). However, there were no associations with other pre-operative characteristics, such as gender, serum total bilirubin, Child-Pugh class, ALBI grade, model for end-stage liver disease (MELD) score, antiviral treatment, nucleos(t)ide analogues, liver cirrhosis, tumor number, vein invasion, and histology grade, and types of surgery.

**Table 4:** Comparison of clinical and pathological characteristics between patients with ALBI grade I and II/III after 1 year of curative resection.

	ALBI grade I (n = 171)	ALBI grade II/III (n = 66)	P value
Age (years; mean ± SD)	52.4 ± 10.4	56.2 ± 12.3	0.019
Age (>60 years), n (%)	48 (28.1%)	35 (53.0%)	<0.001
Male, n (%)	150 (87.7%)	56 (84.8%)	0.557
Bilirubin (g/dL; mean ± SD)	0.8 ± 0.3	0.8 ± 0.3	0.755
Albumin (g/dL; mean ± SD)	3.9 ± 0.6	3.7 ± 0.5	0.038
AFP (>15 ng/mL), n (%)	94 (55.6%)	30 (46.9%)	0.232
Liver cirrhosis, n (%)	48 (49.1%)	37 (56.1%)	0.338
NAs treatment, n (%)	63 (37.3%)	21 (32.3%)	0.635
LAM, n (%)	8 (12.7%)	5 (23.8%)	
LdT, n (%)	2 (3.2%)	1 (4.8%)	
ETV, n (%)	45 (71.4%)	13 (61.9%)	
TDF, n (%)	8 (12.7%)	2 (9.5%)	
Tumor number (single: multiple)	9: 162	2: 64	0.464
Types of surgery			0.818
Right lobectomy, n (%)	24 (14.2%)	10 (15.4%)	
Left lobectomy, n (%)	19 (11.2%)	9 (13.8%)	
Segmentectomy, n (%)	126 (74.6%)	46 (70.8%)	
Child-Pugh grade (A: B)	163: 8	60: 6	0.197
MELD score (mean ± SD)	8.0 ± 2.6	7.7 ± 3.2	0.496
ALBI grade (I: II)	80: 89	25: 40	0.221
Diabetes Mellitus	27 (15.8%)	18 (27.3%)	0.043
Micro/Macrovascular invasion, n (%)	75 (43.9%)	32 (48.5%)	0.521
pTNM stage (I: II: III)	88: 78: 5	31: 27: 8	0.021
Histological grade (well: moderate: poor)	23: 141: 7	10: 53: 3	0.928

AFP = α-fetoprotein, NAs, = nucleot(s)ide analogues, LAM = lamivudine, LdT = telbivudine, ETV = entecavir, TDF = tenofovir, MELD = model for end-stage liver disease, FIB-4 = fibrosis-4, ALBI = albumin-bilirubin.

## Discussion

Liver resection remains the most effective treatment currently for patients with hepatocellular carcinoma, but the long-term prognosis after hepatectomy for HCC is still unsatisfactory due to a high intrahepatic recurrence rate^1,9^. Our study demonstrated that the ALBI grade at the first year post-operation predicts the prognosis of HCC patients after liver resection. The ALBI grade has currently been proven as an objective, evidence-based tool for assessing liver function^8,10,11^. It has also been proposed as a tool to predict the OS among HCC patients after curative liver resection^8,12,13^. The studies conducted were based on the baseline or pre-operative ALBI grade. Consistent with previous reports, our study also demonstrated that a high grade preoperative ALBI grade correlates with poor OS, but not with HCC recurrence.

In the present study, 57% (301/525) of patients were classified as grade I, and the other patients were classified as grade II (43%, 224/525). The liver-related survival rate of the group with ALBI grade I was higher than that of the group with grade II (p = 0.035). Since liver function can change over time after liver resection, it is uncertain whether the pre-operative ALBI grade is better than the post-operative ALBI grade. We found that the ALBI grade at one year after hepatectomy was an independent predictor for HCC recurrence and OS. The liver function after liver resection is an important element for outcomes, and the ALBI grade has been proposed and validated for the assessment of liver function and failure.

In this study, we chose the first year after resection as our second time to evaluate the impact of ALBI grade after resection. If we chose a later second time, such as the second or third year after resection, there might be more patients with recurrent HCC before the second time who would be excluded from the RSF analysis. In contrast, if we chose an earlier time, such as the third or sixth month after resection, the liver function might not be recovering completely. Second, Chen *et al*.^14^ evaluated the liver regeneration by serial abdominal CT and revealed complete regeneration took about one year. Previous studies also indicated liver regeneration wound continue up to one year after hepatectomy^15–20^. Therefore, we chose the time point of the assessment of post-OP ALBI at one year after resection for comparison.

Based on the results of this study, we recommend using the ALBI grade at the first year post-operation as a significant predictor and checking it routinely in HCC patients after liver resection to predict recurrence and OS. In the future, a prospective study is needed to check serial serum data, including albumin and bilirubin levels, to get the serial ALBI grade after HCC resection to evaluate the precise time to check ALBI grade for HCC recurrence prediction.

Liver function may be influenced by numerous factors, such as tumor burden, reserved liver volume, nutritional status, and post-operative complications. The ALBI grade is determined with albumin and bilirubin. Theoretically, liver function should be recovered after HCC resection. However, in the present study, there were 28 patients with ALBI grade deterioration and 65 patients with ALBI grades II and III after one year of surgery. The pre-operative ALBI grade and post-operative ALBI grade at one year had statistically significant correlation (p < 0.05) (Table S2), but the multivariate analysis indicated that the ALBI grade after liver resection, not the pre-operative ALB grade, was an independent risk factor for RFS and liver-related survival. Hence, we supposed that the pre-operative ALBI grade is affected by the tumor burden, resulting in minimizing the predictive effect. After HCC resection, the post-operative ALBI grade can reflect the environment of the liver exactly to provide more reliable predictive value for RFS and OS without tumor burden. In addition, basic researches showed that albumin suppresses the proliferation and growth of HCC cell through the modulation of AFP or effects on growth-controlling kinases^21,22^. The inhibitory role of albumin on HCC growth may explain why a high ALBI grade occurs and contribute to the recurrence HCC. But, the exact mechanism between liver function, as indicated by the ALBI grade, and tumor recurrence remains unclear and need further study.

It is an unexpected result that only 50.8% of patients in ALBI grade II had cirrhosis. We might explain this phenomenon is due to multiple factors, such as tumor, nutrition, and comorbidity, such as sarcopenia, not only by liver cirrhosis. By this view, ALBI grade is a useful index for pre-operative evaluation. Furthermore, we found that after curative resection, around 70% of patients with pre-operative ALBI grade II improved their ALBI grade (Table S3). Therefore, we suppose that the preoperative ALBI grade might be partially affected by the tumor. Previous studies indicates that hypoalbuminemia is associated with new cancer diagnosis^23^ and advanced cancers^24^. Hence, cancers reduce circulating albumin due to metabolic and vascular effect of tumor and may cause hypoalbuminemia^25^. After tumor resection, the post-operative ALBI grade can reflect the environment of the liver exactly to provide a more reliable predictive effect in RFS and OS without tumor burden. Therefore, we can conclude that the post-operative ALBI grade is much more important and useful than the pre-operative ALBI grade.

A previous study indicated that the ALBI grade has a significant better performance in predicting the outcomes of HCC patients after liver resection than the Child-Pugh classification^26^. The Child-Pugh classification was designed to evaluate the prognosis and outcome of cirrhotic patients. It is now the most widely used method to assess the preserved liver function in HCC patients for further systematic management of HCC^9,27^. However, whether the Child-Pugh classification is appropriate for evaluating the liver function in HCC patients remains to be determined due to patients in the same Child-Pugh classification could be separated into different ALBI grade and have survival difference with the wide range of hepatic reserve within a single Child-Pugh classification. Furthermore, the evaluation of ascites and encephalopathy is highly subjective and may greatly reduce the accuracy of the assessment. In the present study, the majority (96%) of early HCC patients were Child-Pugh class A. In contrast, with the classification of the ALBI grade, 220 (43.7%) patients and 283 (56.3%) patients were stratified into ALBI grades I and II, respectively. Although, the mean ALBI score increases with higher Child Pugh score in our study and the distribution has linear trend (p = 0.004). (Figure S2) The areas under the receiver operating characteristic curve (AUCs) of the ALBI grades for postoperative recurrence and mortality are significantly higher than that of the Child-Pugh classification. This result is compatible with previous studies. In the future, HCC management should seriously consider integrating the ALBI grade into the stratification of hepatic function among patients with resectable HCC.

With regard to anti-HBV therapy, previous studies showed that nucleoside analogue treatment would reduce the recurrence of HCC after tumor resection^28,29^. However, some studies indicated the opposite findings^30^. In our current study, there was no association between HBV treatment, survival and recurrence free survival after hepatectomy. However, the result is not very solid because of the diversity and complexity of antiviral treatment within our study cohort. The National Health Insurance in Taiwan covers the treatment of HBV, but patients with hepatocellular carcinoma are not supported only when patients had liver cirrhosis with serum HBV DNA > 2000 IU/mL. As a result, many patients are treated with self-paid nucleos(t)ide analogues(NAs), which leads to poor compliance. Furthermore, in the earlier era of HBV treatment with lamivudine and adefovir, HBV DNA measurements were not very popular in clinical practice, which induced virological resistance without early detection and the change of drugs. Hence, the evidence is too weak to conclude that the usage of nucleos(t)ide analogues(NAs) is not associated with RFS and OS. However, we have still seen better trends of RFS and OS in more recent times (2009-2016) compared with the early era (2001-2008), although the changes in either one are not significant (Figure S1). Furthermore, there is no statistically significance in RFS and OS by different NAs (Figure S3).

Aside from the ALBI grade, we also showed that age, DM, AFP, platelet count, and liver cirrhosis are important predictors of HCC recurrence. Furthermore, age, DM, AFP, and TNM stage were independent risk factors for OS. This is consistent with the results of previous studies in which patient factors (age, DM, and platelet count)^31–33^, liver background factors (liver cirrhosis)^34^, and tumor factors (TNM stage, and AFP)^35^ contributed to the outcomes of HCC patients.

In the present study, there are 253 patients with recurrence, 19 patients received hepatectomy, 81 patients received radiofrequency ablation (RFA), 114 patients underwent transcatheter arterial chemoembolization (TACE), 4 patients received TACE and RFA concomitantly, 6 patients received percutaneous ethanol injection (PEI), 14 patient received systemic treatment (target therapy or palliative chemotherapy), 15 patients chose hospice care. The ALBI grade at recurrence might affect the choice of treatment and have different prognosis. In our study, different treatment strategies have diverse ALBI grade distribution. In hospice group, ALBI grade III is up to 41.7%. (Figure S4) The overall survival result revealed those who received resection or RFA had better outcomes than patients receive TACE then follows with TACE/RFA, PEI and the group who received systemic treatment or hospice care.(Figure S5 and Table S1) Plans of retreatment is based on tumor size, numbers, lymph nodes involvement and liver function reservation once recurrence. Therefore, if we can detect the recurrence earlier with preserved liver function, the following treatment plan and the outcome will be better. Post-operative ALBI grade provides the evaluation of liver reservation and would be a useful model for early prediction of HCC recurrence.

There are some limitations in our study. First, the information and data are retrospectively collected from medical records. Some patients didn’t return to our hospital for further follow-up or even died after the operation. Therefore, some important data was insufficient, such as complete serum bilirubin and albumin levels. The precise time of ALBI grade for prognosis evaluation needs prospective study for further assessment. Second, referral bias could not be completely avoided due to all patients in our study cohort were treated at a tertiary medical center. In addition, although we indicated that ALBI grade variation after hepatectomy contributes significantly to the accuracy of OS prediction, the generalizability of the change in ALBI grade requires further analyses to evaluate its survival impact.

In summary, our present study demonstrated that the post-operative ALBI grade at one year after resection is a valuable serum index for assessing the recurrence and survival of HCC patients undergoing hepatectomy. Nevertheless, further researches are needed before applying ALBI grade into daily practice.

## Patients and methods

### Patients

We reviewed a total of 2195 patients who were diagnosed with HCC and underwent surgical resection between April 2001 and March 2017 at Kaohsiung Chang Gung Memorial Hospital. This hospital is a tertiary referral center that covers the southern part of Taiwan. We excluded 479 patients with prior HCC treatment, 543 patients with HCV infection, 97 patients with coinfection with HBV and HCV, 97 patients without HBV or HCV infections, and 186 patients who underwent liver transplantation. Ultimately, we recruited 525 chronic hepatitis B (CHB) patients with HCC who underwent primary curative resection.

HCC was defined according to the results of imaging studies and biochemical assays, and the diagnosis was confirmed using histopathology. The HCC diagnosis was based on the criteria of the practice guidelines of the European Association for the Study of the Liver (EASL) or the American Association for the Study of Liver Disease (AASLD)^36,37^. This study complies with the standards of the Declaration of Helsinki and current ethical guidelines, and approval was obtained from the Ethics Committee of Chang Gung Memorial Hospital. Written informed consent was obtained from all patients.

### Assessments and follow-up evaluation

The baseline demographics, serum biochemistry, tumor burden and antiviral therapy were comprehensively recorded before any forms of definite treatment. Types of surgery were categorized into right lobectomy, left lobectomy, and segmentectomy. The diagnosis of cirrhosis was documented by resected non-tumor pathologic report. The HCC stage was defined according to the Barcelona Clinic Liver Cancer (BCLC) guidelines. Tumor differentiation was determined using the Edmondson grading system. The follow-up ended on December 31, 2018. OS was defined as the interval between the dates of surgery and death or last observation. Patients were followed up at the 1^st^ month after liver resection, followed by every 3 months in the first year and every 3-6 months in subsequent years. Routine tests such as serum AFP levels, serum biochemistry, abdominal ultrasound were performed at every follow-up. Abdominal computed tomography or magnetic resonance image were performed at the 1^st^ month after liver section and every 12 months or recurrence is suspected clinically. Patients with relapses were treated with liver resection, radiofrequency ablation (RFA), transcatheter arterial chemoembolization (TACE), percutaneous ethanol injection, or sorafenib, depending on liver functional status, extent of disease, and performance and economic status.

### Calculation of ALBI grades

The ALBI scores were calculated as follows: linear predictor = (log10 bilirubin × 0.66) + (albumin × −0.085), where the units of bilirubin and albumin are in units of μmol/l and g/l, respectively. The ALBI grades were stratified into three grades: grade I,≤ − 2.60; grade II,− 2.60 to ≤ −1.39; and grade III, > − 1.39, as reported previously^8^. The ALBI grades at 1 year was calculated using the serum albumin and bilirubin levels examined at 1 year after resection. We classified the variation of the ALBI grade before and after operation into three groups: an improved group (from ALBI grade II to I), a constant group (no change of ALBI grade), and a deteriorated group (from ALBI grade I to II or III or from II to III).

### Calculation of FIB-4

The fibrosis-4(FIB-4) values were calculated automatically using the formula age (years) × AST [U/l]/(platelets [10^9^/l] × (ALT [U/l])^1/2^)^38^, in which the age of the patient was at the time of surgery.

### Statistical analysis

Statistical analyses were performed using SPSS 17.0 software (Chicago, IL, USA) and R. Experimental values of continuous variables are expressed as the mean ± standard error of the mean. The chi-squared test and Donferroni correction are used as appropriate to evaluate the significance of differences in data and multiple comparison in groups. The correlation between factors is evaluated with Spearman’s correlation coefficient. The relationship between recurrence-free survival (RFS), OS, and the ALBI grades were analyzed using Kaplan–Meier survival curves and the log-rank test, and p < 0.050 was considered statistically significant. Factors that were significant in the univariate analysis (p < 0.05) were included in a multivariate analysis using a Cox forward stepwise variable selection process of the estimated OS and RFS.

## Supplementary information

Supplementary information
