# Supplementary material for: Ability of the post-operative ALBI grade to predict the outcomes of hepatocellular carcinoma after curative surgery
Source: Sci Rep. 2020 Apr 29;10:7290. doi: 10.1038/s41598-020-64354-0 (PMC7190718; doi:10.1038/s41598-020-64354-0)
Supplement: Supplementary file 1 — Supplementary information [file 41598_2020_64354_MOESM1_ESM.docx]

**Ability of the post-operative ALBI grade to predict the outcomes of hepatocellular carcinoma after curative surgery**

Wei-Ru Cho^1^, Chao-Hung Hung^1^, Chien-Hung Chen^1^, Chih-Che Lin^2^, Chih-Chi Wang^2^, Yueh-Wei Liu^2^, Yi-Ju Wu^2^, Chee-Chien Yong^2^ , Kuang-Den Chen^4^, Yu-Chieh Tsai^3^, Tsung-Hui Hu^1^, Ming-Chao Tsai^1,5^

^1.^Division of Hepato-Gastroenterology, Department of Internal Medicine, Kaohsiung Chang Gung Memorial Hospital and Chang Gung University College of Medicine, Kaohsiung, Taiwan; ^2.^Liver Transplantation Center and Department of Surgery, Kaohsiung Chang Gung Memorial Hospital and Chang Gung University College of Medicine, Kaohsiung, Taiwan; ^3.^Center for Translational Research in Biomedical Sciences, Liver Transplantation Program and Department of Surgery, Kaohsiung Chang Gung Memorial Hospital and Chang Gung University College of Medicine, Kaohsiung, Taiwan ^4.^Department of Internal Medicine, Kaohsiung Chang Gung Memorial Hospital; ^5.^ Graduate Institute of Clinical Medical Sciences, Chang Gung University College of Medicine, Taiwan

**Correspondence to**

Ming-Chao Tsai

Division of Hepato-Gastroenterology, Department of Internal Medicine, Kaohsiung Chang Gung Memorial Hospital, 123 Ta Pei Road, Kaohsiung, Taiwan

Tel: +886-7-731-7123, ext. 8310; Fax: +886-7-732-2402;

E-mail: tony0779@gmail.com

**Figure S1: Recurrence-free survival (A) and liver-related survival (B) in HCC patients after curative resection according to the operation year**

**(A)**

**
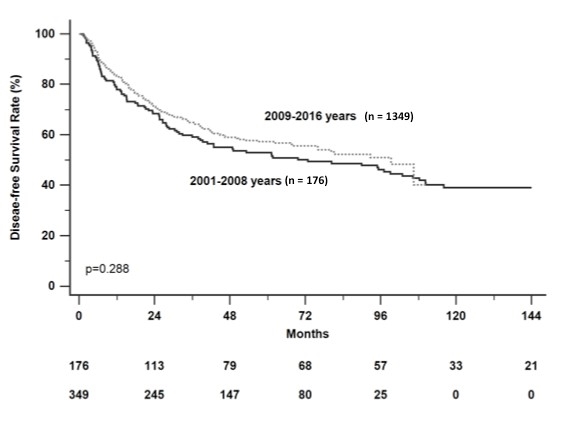
**

**(B)**


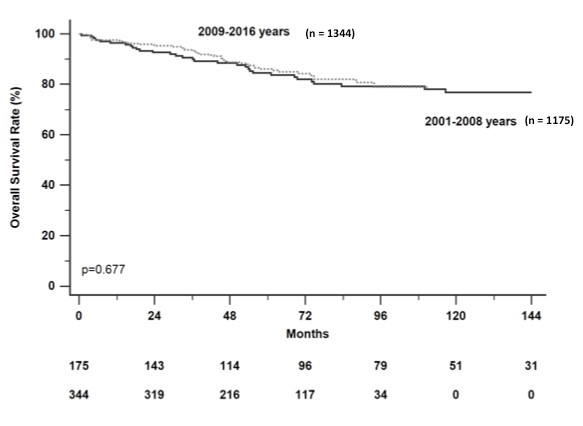


**Figure S2: The distribution of pre- operation Child-Pugh score and pre-operation ALBI score**


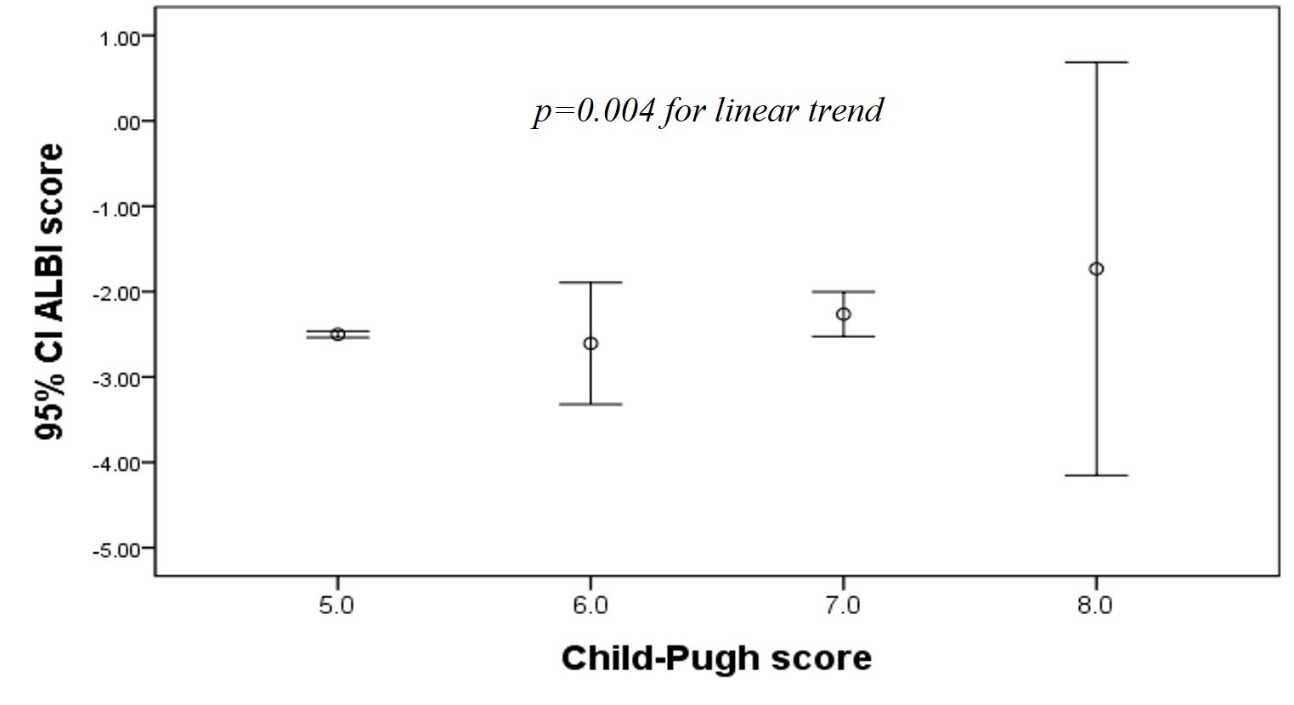


The mean ALBI score increases with higher Child Pugh score (*p=0.004 for linear trend*). Error bars indicate standard deviation.

**Figure S3: Recurrence-free survival (A) and liver-related survival (B) in HCC patients with different anti-HBV therapy after curative resection according to the operation year**

**(A)**

**
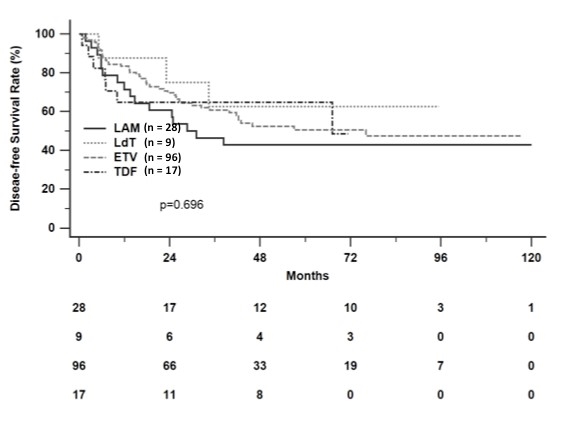
**

**(B)**

**
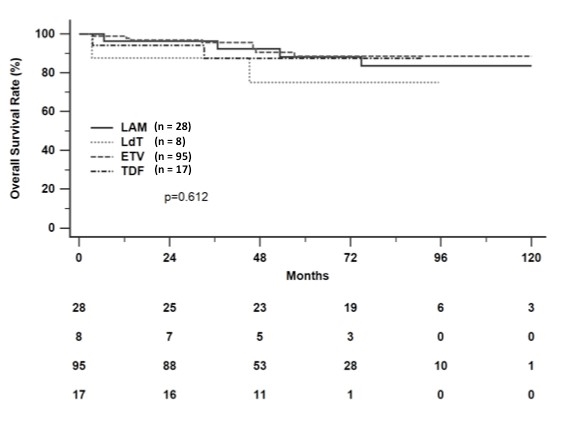
**

LAM = lamivudine, LdT = telbivudine, ETV = entecavir, TDF = tenofovir

**Figure S4: The association between ALBI grade at recurrence and the treatment strategies for recurrence**


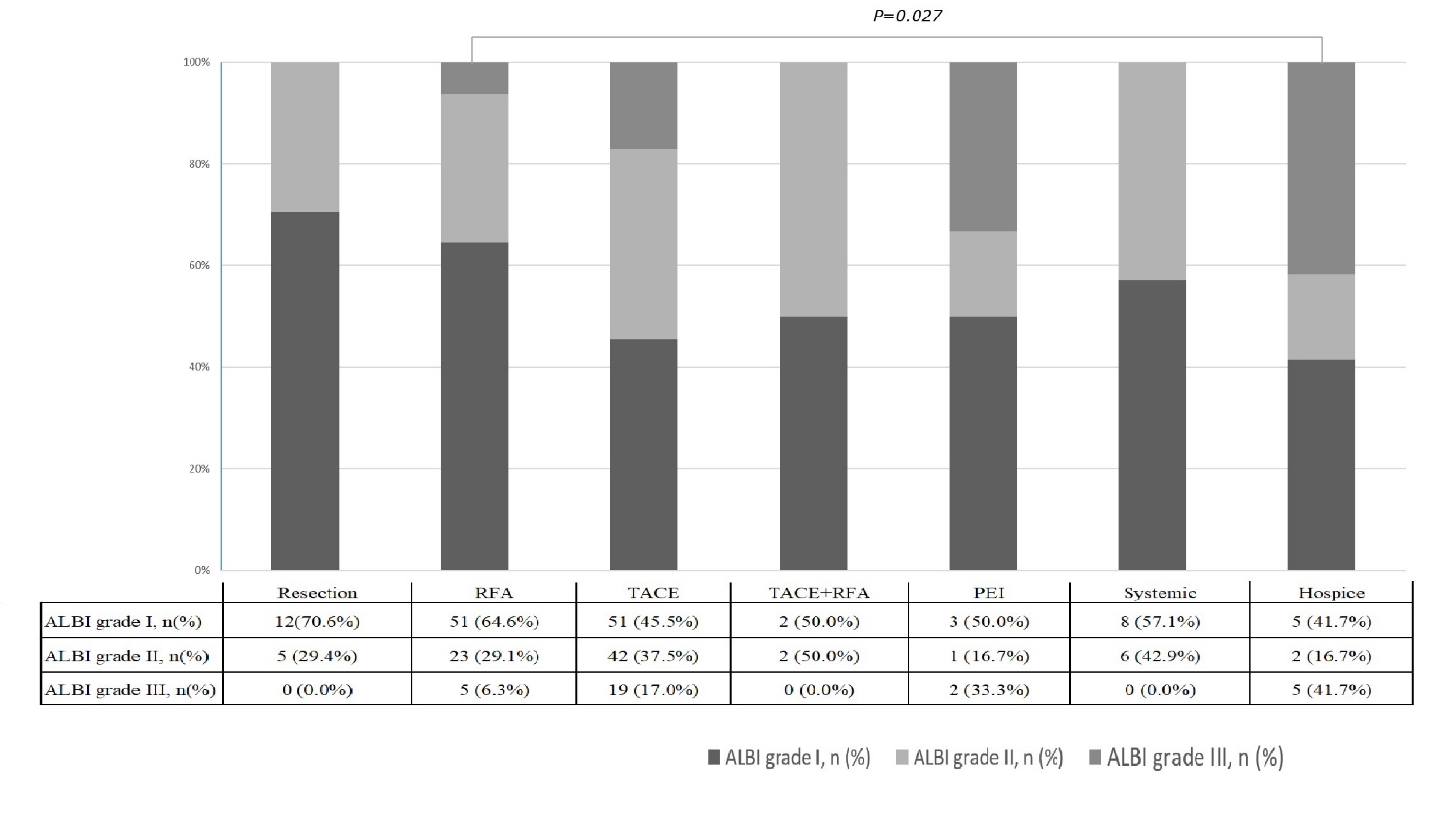


RFA = Radiofrequency ablation, TACE= Trans-arterial chemoembolization, PEI= Percutaneous ethanol injection, ALBI = albumin-bilirubin.

Treatment plans are associated with the different ALBI grades at recurrence. The RFA group has lower percentage of ALBI grade III than in hospice.( *p=0.027*)

Chi square test and Donferroni correction for multiple comparison.

**Figure S5: Kaplan-Meier curves by treatment for recurrences**

**
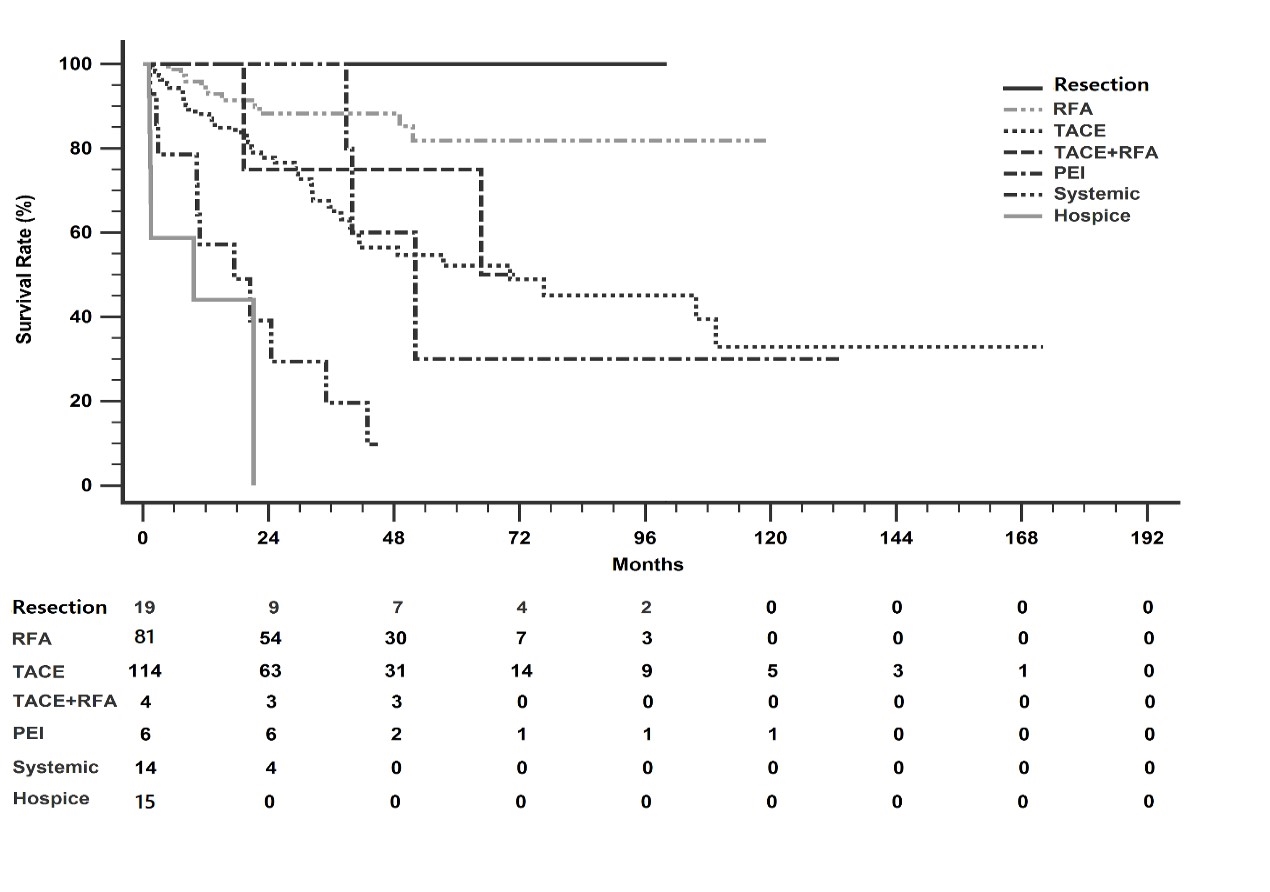
**

**Table S1: P value between treatment at recurrence**

|  | **Resection** | **RFA** | **TACE** | **TACE/RFA** | **PEI** | **Systemic** |
| --- | --- | --- | --- | --- | --- | --- |
| **RFA** | **ns** |  |  |  |  |  |
| TACE | 0.008 | <0.001 |  |  |  |  |
| TACE/RFA | 0.043 | ns | ns |  |  |  |
| PEI | 0.014 | ns | ns | ns |  |  |
| Systemic | <0.001 | <0.001 | <0.001 | 0.045 | 0.013 |  |
| Hospice | <0.001 | <0.001 | <0.001 | ns | 0.007 | ns |

RFA = Radiofrequency ablation, TACE= Trans-arterial chemoembolization, PEI= Percutaneous ethanol injection, ALBI = albumin-bilirubin. *p*>0.05, ns

Treatment with resection or RFA when recurrence have better survival than systemic therapy or hospice care. (*p<0.001*)

**Table S2: The correlations between pre-OP serum albumin levels and pre-OP ALBI score, and post-OP 1 year ALBI score and post-OP 1 year serum albumin levels**

| Spearman’s correlation coefficient | pre-OP albumin | pre-OP ALBI score | post-OP albumin | post-OP ALBI score |
| --- | --- | --- | --- | --- |
| pre-OP albumin | 1 | -0.969** | 0.112 | -0.114 |
| pre-OP ALBI score |  | 1 | -0.108 | 0.142* |
| post-OP albumin |  |  | 1 | -0.938** |
| post-OP ALBI score |  |  |  | 1 |

The inverse correlation between pre-operation albumin and pre-operation ALBI score, post-operation albumin and post-operation ALBI score are significant (p<0.001). Spearman’s correlation coefficient. Statistical significance indicated by * (p < 0.05), **(p < 0.001)

**Table S3 Association between pre- and post-operative ALBI grades**

|  |  | Pre-OP ALBI Grade I | Pre-OP ALBI Grade II | Total |
| --- | --- | --- | --- | --- |
| Post-operative first year | ALBI Grade I | 80 (76.2%) | 89 (69.0%) | 169 (72.2%) |
|  | ALBI Grade II | 24 (22.9%) | 37 (28.7%) | 61 (26.0%) |
|  | ALBI Grade III | 1 (1.0%) | 3 (2.3%) | 4 (1.3%) |
|  |  | 105 | 129 | 234 |
